# Widely Targeted Metabolomics Analysis Reveals Great Changes in Nonvolatile Metabolites of Oolong Teas during Long-Term Storage

**DOI:** 10.3390/molecules26237278

**Published:** 2021-11-30

**Authors:** Cuiyun Hong, Wenjie Yue, Qingyu Shen, Wenhua Wang, Hongyan Meng, Ying Guo, Wenjiang Xu, Yaling Guo

**Affiliations:** 1 Fujian Key Laboratory of Subtropical Plant Physiology and Biochemistry, Fujian Institute of Subtropical Botany, Xiamen 361006, China; hongcy1983@163.com (C.H.); 13906020193@163.com (Q.S.); wangwenhua0629@163.com (W.W.); mhy1984@126.com (H.M.); bibygylc@163.com (Y.G.); xumpa@sina.com (W.X.); 2 Jinshan College, Fujian Agriculture and Forestry University, Fuzhou 350002, China; ajie775@sina.com; 3 College of Horticulture, Fujian Agriculture and Forestry University, Fuzhou 350002, China

**Keywords:** chemical constituents, store time, oolong tea, targeted metabolomics

## Abstract

As a semifermented tea, oolong is exceedingly popular worldwide for its elegant, flowery aroma and mellow, rich taste. However, recent marketing trends for old oolong teas and their chemical quality largely remain unexplored. In this study, we applied widely targeted metabolomics using ultra-performance liquid chromatography-tandem mass spectrometry (UPLC-MS/MS) combined with multivariate analysis to investigate the chemical change of oolong teas in the aging process. With the increasing of store time, most nongalloylated catechins; tannins, including TFs and proanthocyanidins; flavonols and glycosylated flavonols; amino acids and their derivatives; nucleotides and their derivatives; and lots of alkaloids and phospholipids declined, while most fatty acids and organic acids increased, and galloylated catechins, GA, and caffeine were almost stable. The result also suggested that approximately seven years (but not an infinite extension) was a special period for oolong tea storage, which brings about excellent taste.

## 1. Introduction

Tea is one of the most widely consumed beverages worldwide. As a typical “semifermented” tea, oolong tea is very famous for its elegant, flowery aroma and mellow, rich taste. Among all kinds of tea, the processing technology of oolong tea is the most complicated, which includes fresh leaves’ harvesting, solar/indoor withering, turning over/setting, firing, rolling, and drying [1]. The term “semifermented” refers to an enzymatic oxidation process in the post-harvest manufacturing practice, with a degree of fermentation between green (unfermented) and black tea (fully fermented). The final fermented degree of oolong tea usually ranges from 20 to 80% [2].

Sometimes, aging is crucial for the quality improvement of food, during which microbial fermentation, enzymatic hydrolysis, or oxidation may occur [3]. There is a deep belief in China that aging can improve the quality of white tea and dark tea (Pu-er tea). However, recently, old oolong teas became increasingly prevalent in the consumer market, especially in Fujian, Guangdong, and Taiwan, the main producing areas of oolong tea. People believe that the longer oolong teas are stored and further oxidized gradually, the better they are in terms of taste and health benefits. Oolong teas used to develop to an outstanding, old oolong tea usually need appropriate management, such as a fierce baking treatment at the beginning but without other measures in the following long-term storage, or periodical baking refinement in a specialized oven at various temperatures between 100 and 140 °C [2]. Several studies on oolong tea with a long storage period have been carried out, which may help us to understand the related chemicals of sensory change and health efficiency. For instance, Lee et al. [4,5] found a massive accumulation of gallic acid and the unique occurrence of myricetin, quercetin, and kaempferol in preparing old oolong teas and revealed that gallic acid was released from the dimer of EGCG, not directly degraded from EGCG. Chen et al. [2] showed that the contents of phenolic compounds in old Tieguanyin oolong teas were altered by the baking but remained relatively stable thereafter in the aging process, while the contents of major catechins derivatives and flavonol glycosides reduced. Wang et al. [6] even suggested that the relative content of gallic acid over 5-galloylquinic acid could be used as an index for the baking intensity of oolong teas, which was found in preparing old oolong tea by baking annually. In addition, the biochemical composition of three varieties of old oolong teas stored in 1990 and 2016 and their hyperglycemic and hypolipidemic activities in vitro were compared by Hou et al. [7], which demonstrated that the contents of total polyphenols, catechins, and amino acids of all three varieties of old oolong teas stored in 2016 and their activities of inhibiting α-amylase and pancreatic lipase were higher than that in 1990, but the opposite was true for the content of gallic acid. These previous studies mainly focused on polyphenolic compounds' analysis, especially catechins and gallic acids, rather than other metabolites, and were based on baking periodically during storage.

The current study mainly aimed at analyzing the chemical change of oolong teas refined completely without other measures such as baking periodically in the following long-term storage, due to most ordinary consumers lacking the skills and facility to carry out professional baking. It is recognized that taste is a crucial quality index of Camellia teas [8], which is directly affected by the chemical composition of compounds such as catechins, flavonoids, amino acids, tannins, purine alkaloids, and others, and their contents and proportions [9]. With an increasing application of metabolomics in food science, many compounds in food can be accurately quantified [10]. Similarly, metabolomics was exploited to identify the tea cultivar, origin, processing, grade information, etc., in tea research [1,11,12,13]. In this study, instead of using conventional chemical analysis, we employed ultra-performance liquid chromatography-tandem mass spectrometry (UPLC-MS/MS) based on widely targeted metabolomics analysis and purposely chose the typical premium cultivar, Camellia sinensis cv. Shuixian., which is identified as a national variety by the Chinese National Crop Cultivar Certification Committee [14,15] and a predominant cultivar of Wuyi rock tea, to decipher the metabolic profiling change of oolong teas in the aging process.

## 2. Results and Discussion

### 2.1. Nonvolatile Metabolite Profile of Oolong Teas with Storage Age

To study the change of nonvolatile metabolites of oolong teas, widely targeted metabolic analysis was applied to profile methanol-soluble extraction of tea samples which have been stored for 1, 3, 7, 14, and 25 years. Considering the different polarities of boiling water and organic solvents and the differences in the extraction efficiency for various metabolites in tea, the 70% aqueous methanol solution was chosen as the extraction solvent to extract more abundant metabolites [16,17]. Then, a total of 591 metabolites were identified (Table S1), including 147 flavonoids, 33 tannins, 80 phenolic acids, 50 amino acids and derivatives, 40 nucleotides and derivatives, 26 alkaloids, 40 organic acids, 103 lipids, and others.

**Figure 1:**
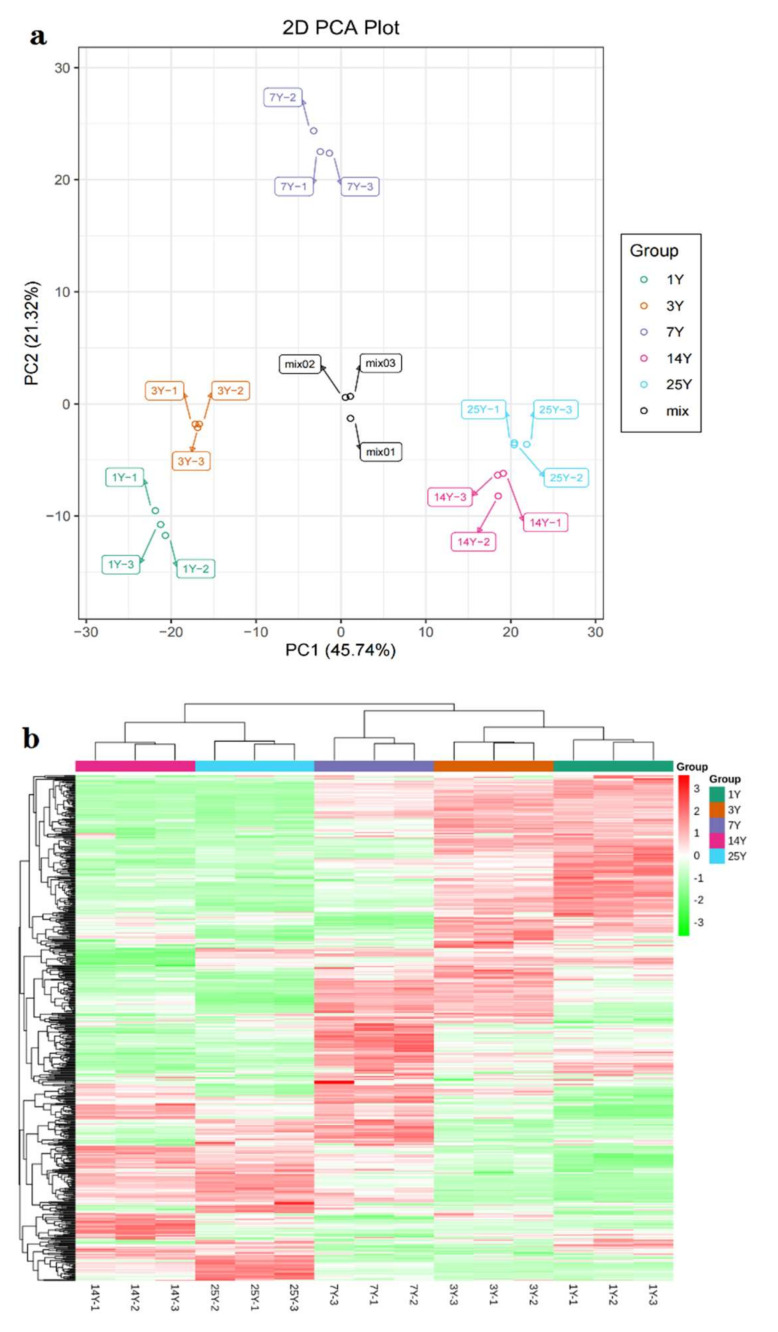
PCA score plot (**a**) and heat map (**b**) of the metabolites in oolong teas stored for 1 y, 3 y, 7 y, 14 y, and 25 y. Red represents high contents, while green represents low contents (ranges from −3.0 to 3.0).

According to these 591 metabolites, principal component analysis (PCA) was demonstrated to visualize the similarity of the samples [18]. As shown in Figure 1a, the first principal components of PCA explained 45.74%, accompanied by the second principal components influencing 21.32% of the variance. There were clear separations of samples among different storage and close clusters of the same storage year, i.e., 1 y, 3 y, 7 y, and 14 y, and 25 y. In the PCA score plot, the first principal components of these five groups were orderly distributed from negative to positive on the X-axis, indicating that the nonvolatile changes paralleled the length of storage duration. On the Y-axis, a parabolic pattern with 7 y on top of the curve, while 1 y on one tail end and 25 y on the other was evident, which probably meant that seven years of storage could be a special period for oolong teas. It appeared that both 14 y and 25 y were similar, which indicated the transformation of chemicals in oolong teas tended to be tardy and inactive after long-term storage. 

To further verify metabolite cluster patterns among five groups of tea with different storage periods, a hierarchical clustering analysis (HCA) was also performed. As shown in Figure 1b, 591 metabolites showed that samples were clustered by storage duration, 1 y and 3 y formed a cluster, following clustered with 7 y, and 14 y and 25 y closely clustered. Thus, the HCA result was in accordance with that of PCA. The metabolic profiles of these samples illustrated significant variations in nonvolatiles in relation to storage duration. 

### 2.2. Metabolite Changes in Paired Comparisons

**Figure 2:**
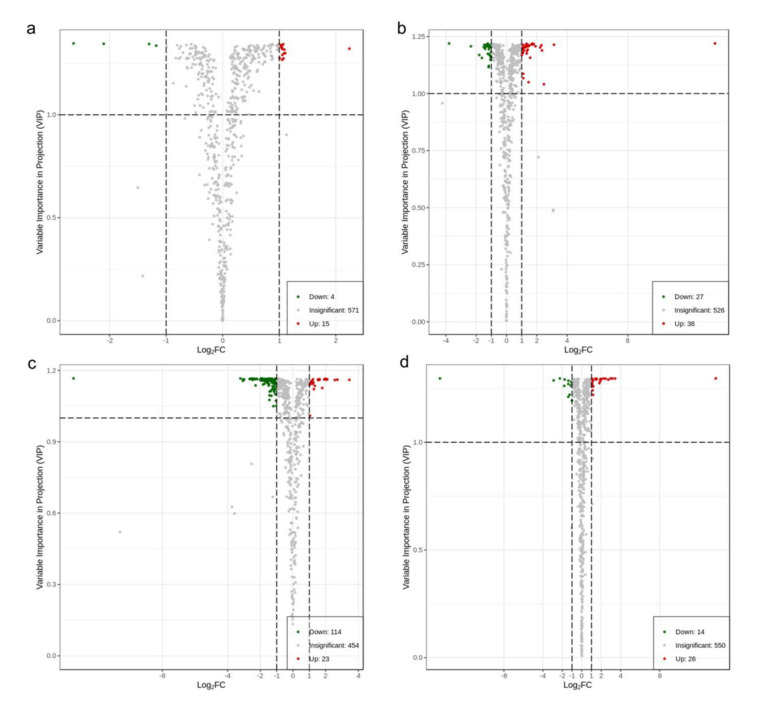
Metabolites with VIP values >1 and fold change ≥2 or 0.5, were selected as differential metabolites for each paired comparison between groups with different storage times. The X-axis was the logarithm of fold change, and the Y-axis was the VIP value. Red represents upregulated metabolites, while green represents downregulated metabolites, and grey represents insignificant metabolites. (**a**): 1 y and 3 y; (**b**): 3 y and 7 y; (**c**): 7 y and 14 y; (**d**): 14 y and 25 y.

**Figure 3:**
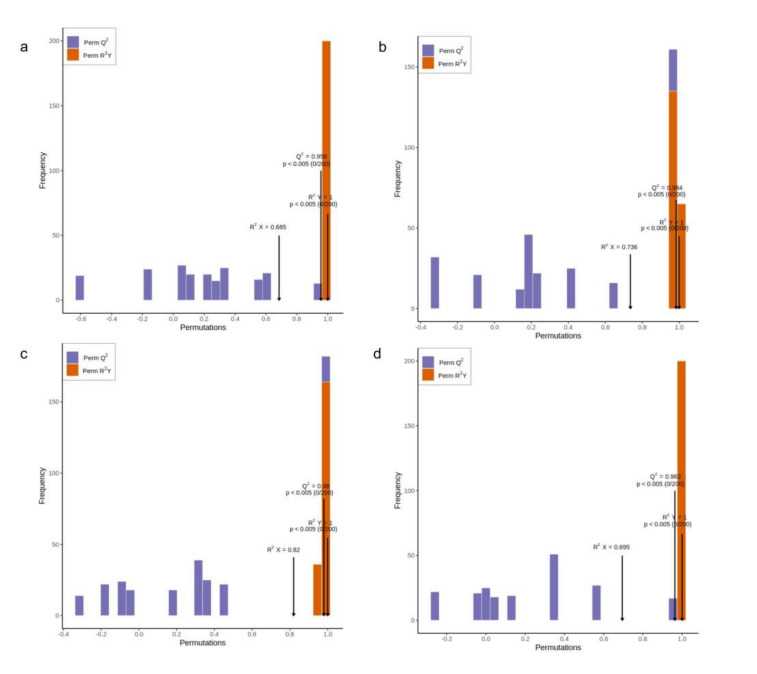
Related permutation tests in OPLS-DA. The X-axis represents the accuracy of the model, and the Y-axis represents the frequency of model classification effect. (**a**): 1 y and 3 y; (**b**): 3 y and 7 y; (**c**): 7 y and 14 y; (**d**): 14 y and 25 y.

To refine the statistical analysis for dramatically changed metabolites, the variable influence on projection (VIP) values based on orthogonal partial least-squares discriminant analysis (OPLS-DA) and fold change with two as the base of the logarithm of all metabolites in paired comparisons were calculated. Any metabolite with VIP values ≥1, which means its corresponding metabolite contributed significantly to the separation of sample groups [19], and fold change ≥2 or ≤0.5, was selected as differential metabolites for each paired comparison between contiguous groups with different storage duration (Figure 2). Their corresponding permutation tests in OPLS-DA were conducted to assess model fitting by iteration 200 times before this. As shown in Figure 3, all the *p* values of Perm Q^2^ and Perm R^2^Y were <0.005, suggesting that there was no over-fitting in those four models. In paired comparison, for instance, in 1 y with a 3 y comparison, the metabolites in 1 y, as a control, were compared with those from the 3 y group. Differential metabolites were upregulated, as well as downregulated. In the 1 y with a 3 y comparison (Figure 2a), there were 15 upregulated (including 12 lipids) and 4 downregulated metabolites, 38 upregulated and 27 downregulated metabolites in the 3 y with a 7 y comparison (Figure 2b), 23 upregulated and 114 downregulated metabolites in the 7 y with a 14 y comparison (Figure 2c), and 26 upregulated and 14 downregulated metabolites in the 14 y with a 25 y comparison (Figure 2d). Within the first seven years of storage, the amounts of upregulated metabolites gradually increased, exceeding the amounts downregulated. However, after seven years of storage, the downregulated metabolites sharply increased, accompanied by the most downregulated metabolites found at 7 y compared to 14 y, which were far more than those for the other groups. The abundance of substances in teas is an important, influential factor for their quality. Thus, it seemed that seven years was a turning point for oolong tea storage and did not encourage the chemical conversion far beyond seven years, as an infinitely extended time would bring about declines in desirable nonvolatile constituents, which was also in accordance with the PCA result.

### 2.3. Changes of Important Metabolites during Storage by K-Means Clustering Analysis

**Figure 4:**
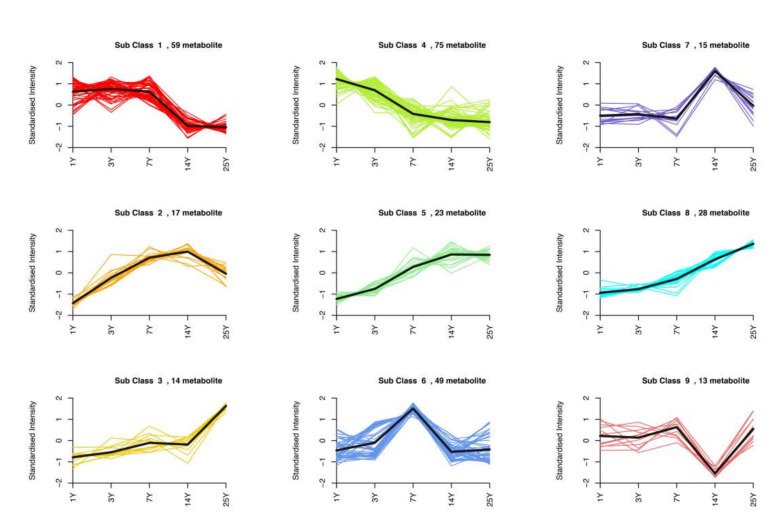
A total of 293 differential metabolites were classified into nine types by K-means clustering (K-means) analysis, based on the variation tendency along with the extent of storage time. The X-axis represents the sample groups, and the Y-axis represents the relative content of metabolites standardized. Subclass represents different change tendencies and the number of metabolites under them.

**Figure 5:**
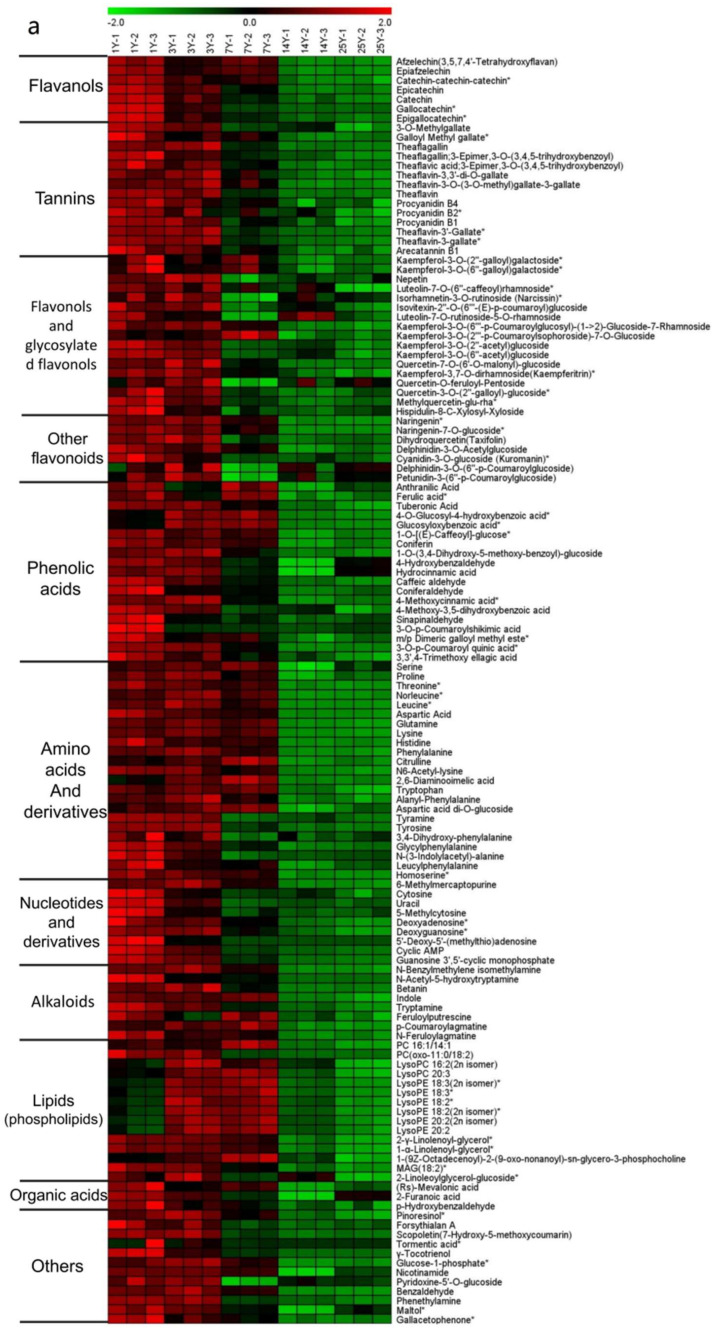

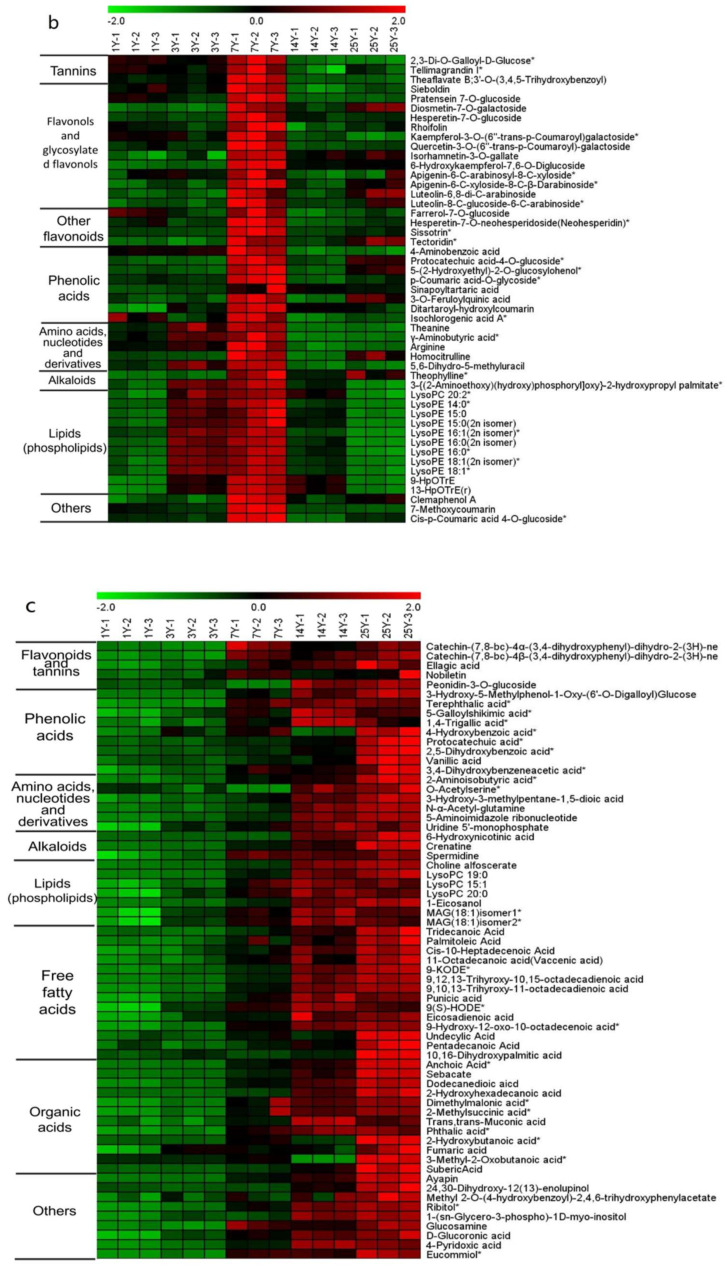
The heat map of differential metabolites which had a similar change tendency by K-means analysis. Red represents high contents, while green represents low contents (ranges from −3.0 to 3.0). “*” means there are isomers existing probably. (**a**): Metabolites in decreasing tendency with storage time extended; (**b**): metabolites which were detected at their highest levels in 7 y samples; (**c**): metabolites in increasing tendency with storage time extended.

To study the variation trend of the relative content of metabolites in different groups, the relative content of differential metabolites was standardized and centralized, and then a K-means clustering (K-means) analysis was performed. A total of 293 differential metabolites were classified into nine types based on the variation tendency, along with the extent of storage duration (Figure 4). According to Figure 4, Cluster 1 and Cluster 4, of a total of 134 substances (Figure 5a), including 31 flavonoids, 19 phenolic acids, 14 tannins, 23 amino acids and derivatives, 9 nucleotides and derivatives, 8 alkaloids, and 15 lipids showed that, as a whole, there was a decreasing tendency with storage time, while Cluster 1 showed more stability in the first seven years than Cluster 4. Meanwhile, Clusters 3, 5, and 8, with a total of 65 compounds (Figure 5c), all demonstrated an increasing tendency upon prolonged storage, which mainly comprised 9 phenolic acids, 21 lipids, 12 organic acids, and others. Substances in Cluster 3 changed placidly in the first 14 years, then sharply after storage, whereas Cluster 5 varied in stability from 14 to 25 years, and Cluster 8 showed a uniform, increasing curve overall. It was also interesting to find that the contents of some important substances in Cluster 6 (49 metabolites, Figure 5b), such as theophylline, L-theanine, γ-aminobutyric acid, isochlorogenic acid A, and a total of 17 flavonoids, 20 tannins, and phenolic acids were at their highest levels in the 7 y samples.

#### 2.3.1. Flavanols and Tannins

Flavanols are the most abundant compounds of tea flavonoids, accounting for 12–24% of the dry tea weights and 70–80% of the total flavonoids [20]. The major compounds of flavanols are tea catechins, which are mainly related to the bitterness and astringency of tea’s taste. During the semifermentation of oolong tea, polyphenol oxidase in the tea leaves catalyzes the oxidation of some catechins into theaflavins [21]. In our current study, it was of interest to find that the contents of most nongalloylated catechins (Figure 5a), such as catechin (C), epicatechin (EC), gallocatechin (GC), and epigallocatechin (EGC), significantly declined with the prolonging of the storage of oolong tea, but most galloylated catechins, such as epigallocatechin-3-gallate (EGCG), epicatechin-3-gallate (ECG), gallocatechin-3-gallate (GCG), and catechin-3-gallate (CG), did not change significantly or regularly, suggesting that galloylated catechins were more stable than nongalloylated catechins during long-term storage, which is possibly due to their esterification groups from the hydroxide radical with gallic acid. According to a previous report, the stability of catechins can be significantly influenced by high temperature, pH, oxygen availability, light, or other factors [22]. Both epimerization and degradation could happen under thermal treatment [23]. The hydration reaction from EGCG can occur at high temperatures, resulting in the production of EGC (or GC) and GA [5]. In our study, oolong tea samples were sealed and stored in a continuously controllable environment, which was shady and cool. Hence, little epimerization [24] and hydration happened on galloylated catechins, which made galloylated catechins relatively stable during long-term storage. Additionally, the loss of nongalloylated catechins is mainly attributed to auto-oxidation during storage. However, galloylated catechins were the main catechins accounting for approximately 75% of the total catechins [25], so that the loss of total catechins was not obvious, which was in accordance with the previous study [26].

A similar decreasing tendency was also observed for tannins (Figure 5a) such as proanthocyanidins and theaflavins components (TFs), which included theaflavins, theaflagallin, theaflavin-3'-gallate, theaflavin-3-gallate, theaflavin-3,3'-di-O –gallate, and theaflavin-3-O-(3-O-methyl) gallate-3-gallate. Theaflavins, as a typical oxidation product of flavan-3-ols, has a similar decreasing tendency during storage in white teas, black teas, and dark teas [10,27,28]. Zhao et al. [29] suggested that long-term storage could enhance polyphenols' polymerization, while oxidative degradation or polymerization of TFs with other compounds could produce thearubigins (TRs) and theabrownines (TBs). This change would turn the color of tea infusion brewed from bright orange–yellow of a younger tea to orange–red of an old tea [20].

#### 2.3.2. Flavonols and Phenolic Acids

Flavonols and glycosylated flavonols are the second most abundant compounds of tea flavonoids, comprising 3–4% of the dry tea weights [20], which are also the main contributor to bitterness and astringency [8,25]. In our study, most flavonols and flavonols-O-glycosides (Figure 5a) such as kaempferol and quercetin reduced during the storage period, whereas most flavones-C-glycosides (Figure 5b) reached their highest contents in the 7 y storage. It appeared that there were some differences in stability existing among acylated glycosylated flavonols, O-glycosylated flavonols, and C-glycosylated flavonols during long-term storage.

Phenolic acids are also an important class of flavonoids, playing essential roles in tea taste. Gallic acid (GA) and chlorogenic acid are the most abundant phenolic acids in tea, with 0.5–1.4% and about 0.3% of dry weights, respectively [20]. During storage of oolong teas, some phenolic acids increased (Figure 5c) and others decreased (Figure 5a), and the number of phenolic acids that decreased exceeded those that increased. In addition, gallic acid (GA) changed irregularly. According to the previous report [4], massive accumulation of GA mainly occurs due to the periodic baking treatment in oolong tea storage, while EGCG could degrade in the thermal process, resulting in GA and EGC (GC). Meanwhile, we noticed that the highest levels of chlorogenic acid and iso-chlorogenic acid (Figure 5b) were detected in 7 y samples. Although the change of chlorogenic acid derivatives in samples was not significant, it was demonstrated that it was significantly correlated to better tea taste [12,30]. Zhou et al. [12] reported that the relative contents of chlorogenic acid in tea leaves show Zhengyan > Banyan > Zhou tea of Wuyi rock tea with a significant difference, *p* < 0.01, which was in line with the order of tea quality. Considering the effect of locality on tea quality, people generally recognize that Zhengyan of Wuyi rock tea is the best, Banyan is the second best, and Zhou tea is the worst. Chlorogenic acid was also identified as a chemical driver of high-quality coffee and flavor modulators, able to significantly increase coffee cup score when added at part-per-million levels, despite no flavor being active when tasted on its own [30]. This discovery echoed the result from PCA and metabolite changes in paired-comparisons analysis, which suggested that seven years was possibly a special age for oolong teas, which could be beneficial for the improvement of quality.

#### 2.3.3. Amino Acids and Nucleotides

The umami matter of tea mainly comprises amino acids and derivatives and nucleotides and derivatives, including proteinaceous amino acids such as glutamine acid, serine, proline, and special nonproteinaceous amino acids such as theanine and γ-aminobutyric acid (GABA) [10]. In the present study, most proteinaceous amino acids (Figure 5a), including serine, proline, glutamine, lysine, leucine, tyrosine, and nucleotides and derivatives such as 6-methylmercaptopurine, cytosine, uracil, and deoxyadenosine, reduced as storage age increased. Considering the deactivation of enzymes and reduction in water contents, the hydrolysis of proteins was generally unconcerned in dry tea during storage, but reactions such as Maillard and degradations led to a decrease in amino acids [31]. Meanwhile, both special nonproteinaceous theanine and GABA (Figure 5b) showed an increasing trend during the first seven years, followed by a decreasing trend, with the highest contents in 7 y samples. In nature, glutamic acid and ethylamine are precursors for theanine biosynthesis [32]. In the present study, glutamic acid had a similar change tendency with theanine, and its derivatives (N-Acetyl-glutamic acid and 3-Hydroxy-3-methylpentane-1,5-dioid acid) increasing after seven years, suggesting that the reactions that occurred on amino acids during storage were diversiform and complicated. Huang et al. [27] reported that L-theanine showed a sharp decrease when storing time was over 10 years in Keemun black tea. 

#### 2.3.4. Alkaloids, Fatty Acids, Organic Acids, and Others

Alkaloids, fatty acids, organic acids, and all other chemicals are all important for forming the complex and charming taste of tea. Alkaloids in tea infusion present bitterness, while fatty acids elucidate an aged and slightly sweet taste, and organic acids are usually sour [9,10,27]. In this study, many alkaloids (Figure 5a) such as N-Acetyl-5-hydroxytryptamine, betanin, indole, and tryptamine declined with stored time, while the main purine alkaloids, caffeine and theobromine, showed no significant change, and theophylline (Figure 5b) was detected at its highest level at 7 y samples. Lee et al. [33] reported the effect of storage time and temperature on chemical constituents in green tea, which also demonstrated that the total alkaloids content decreased during long-term storage, but caffeine was almost unchanged in all different storage conditions.

In addition, the loss of phospholipids (Figure 5a) such as LysoPE and LysoPC, and glycerol esters such as 2-γ-linolenoyl-glycerol and 1-α-linolenoyl-glycerol with the time stored was observed, along with an increase in the free fatty acids of chain lengths above 12 (Figure 5c), such as palmitoleic acid, punicic acid, undecylic acid, pentadecanoic acid, vaccenic acid, and others, which suggested that the oxidation or hydrolysis of phospholipids would be the source of the free fatty acids that increased. Stagg et al. [34] also reported that lipid autoxidation in tea could occur at low moisture content and was inhibited by high water activity, and that palmitoleic acid and C18 fatty acids were predominate among the increased fatty acids. It appeared that long-chain fatty acids were more favorable for accumulation, while short-chain fatty acids were present with lower contents due to volatility [28,34].

Among the screened differential metabolites, most organic acids (Figure 5c) showed an increasing tendency with storage time, including 2-hydroxybutanoic acid, fumaric acid, 2-methyl-2-oxobutanoic acid, suberic acid, anchoic acid, sebacate, fumaric acid, and others, although there were a few others (Figure 5a) such as (Rs)-mevalonic acid, 2-furanoic acid, and p-hydroxybenzaldehyde that decreased. Perez-Burillo demonstrated that the increase in some organic acids might partially result from the degradation of Maillard reaction products such as 5-hydroxymethylfurfural (HMF), which frequently occurs during long-term storage of foods [35]. Organic acids in tea infusion could combine with Ca^2+^ in brewing water, inducing tea cream and sediment formation [36], which might make an old tea infusion more turbid.

## 3. Materials and Methods

### 3.1. Experimental Materials 

Oolong teas (C. Sinensis cv. Shuixian) with supreme grade were produced in 2019, 2017, 2013, 2006, and 1995. Each year, several batches of tea samples were processed and packed separately, then sealed and stored in a cellar located at Winexpress Hoodings Limited in JianOu County, Fujian Province, China. Different batches of samples were prepared by the same cultivated tea garden, cultivated variety, and processing parameters. To the date of our sample analysis in 2020, these tea samples were stored for 1, 3, 7, 14, and 25 years, respectively. A total of three technical replicates were prepared for each year.

Liquid-chromatography-grade solvents, methyl alcohol, acetonitrile, and ethyl alcohol were obtained from Merck Company (Darmstadt, Germany), and all other reagents with analytical grade were purchased from BioBiopha (Kunming, China, http://www.biobiopha.com/) or Sigma-Aldrich (Shanghai, China, http://www.sigmaaldrich.com/united-states.html).

### 3.2. Sample Preparation and Extraction

The metabolomic analysis was conducted according to a previously reported method with a little adjustment [16]. The tea samples stored at −20 °C were vacuum freeze-dried in a Scientz-100F freeze dryer (Ningbo Scientz Biotechnology Co., Ltd., Ningbo, China). The freeze-dried samples were crushed using a mixer mill (MM 400, Retsch GmbH, Dűsseldorf, Germany) with zirconia beads for 1.5 min at 30 Hz. A portion of ground powder (100 mg) was weighed, extracted with 1.2 mL of 70% aqueous methanol solution, stored at 4 °C with stirring overnight, then centrifuged at 10,000× *g* for 10 min. The supernatant was absorbed (CNWBOND Carbon-GCB SPE Cartridge, 250 mg, 3 mL; ANPEL, Shanghai, China, http://www.anpel.com.cn/cnw) and filtrated through a 0.22 μm pore filter paper (SCAA-104, 0.22 μm pore size; ANPEL, Shanghai, China, http://www.anpel.com.cn/) prior to UPLC-MS/MS determination.

### 3.3. UPLC Conditions

The sample extracts were analyzed using a UPLC-ESI-MS/MS system (UPLC, Shim-pack UFLC SHIMADZU CBM30A system, http://www.shimadzu.com.cn/, Shimadzu Corporation, Tokyo, Japan; MS, Applied Biosystems 6500 Q TRAP, http://www.appliedbiosystems.com.cn/, AB SCIEX Pet. Ltd, Framingham, MA, USA). The UPLC used an Agilent SB-C18 column (1.8 µm, 2.1 mm * 100 mm), with the mobile Phase A of pure water with 0.1% formic acid and Phase B of acetonitrile. Sample measurements were performed with a gradient program that employed the starting conditions of 95% A and 5% B. Within 9 min, a linear gradient to 5% A, 95% B was programmed, and a composition of 5.0% A and 95% B was kept for 1 min. Subsequently, a composition of 95% A and 5.0% B was adjusted within 1.10 min and kept for 2.9 min. The column oven was set to 40 °C, and the injection volume was 2 μL. The effluent was alternatively connected to an ESI-triple quadrupole-linear ion trap (Q TRAP)-MS.

### 3.4. ESI-Q TRAP-MS/MS

Linear ion trap (LIT) and triple quadrupole (QQQ) scans were acquired on a triple-quadrupole-linear ion trap mass spectrometer (Q TRAP, API 6500 Q TRAP UPLC/MS/MS System, AB SCIEX Pet. Ltd, Framingham, MA, USA), equipped with an ESI Turbo Ion-Spray interface, operating in positive and negative ion mode and controlled using the Analyst 1.6.3 software (AB Sciex, AB SCIEX Pet. Ltd, Framingham, MA, USA). The ESI source operation parameters were as follows: ion source, turbo spray; source temperature, 550; ion spray voltage (IS), 5500 V (positive-ion mode)/−4500 V (negative-ion mode); ion source gas I (GSI), gas II (GSII), curtain gas (CUR) was set at 50, 60, and 30 psi, respectively; the collision gas (CAD) was high. Instrument tuning and mass calibration were performed with 10 and 100 μmol/L polypropylene glycol solutions in QQQ and LIT modes, respectively. QQQ scans were acquired as multiple reaction monitoring (MRM) experiments with collision gas (nitrogen) set to 5 psi. Declustering potential (DP) and collision energy (CE) for individual MRM transitions were completed with further DP and CE optimization [16]. A specific set of MRM transitions were monitored for each period according to the metabolites eluted within this period.

### 3.5. Qualitative and Quantitative Analysis of Metabolites

A self-built database, MetWare database (MWDB, Metware Biotechnology Co., Ltd., Wuhan, China), was mainly used to perform a qualitative analysis of the substances based on secondary spectral information, while the possible redundancy caused by different isotopes; in-source fragmentation; K^+^, Na^+^, and NH^4+^ adduce; and dimerization were removed. Firstly, extracted ion chromatograms (XICs) for each Q1 (*m*/*z* ± 0.2Da) were evaluated for the presence of the targeted substance by analyzing corresponding mass spectra to obtain their accurate (*m*/*z*). Then, for each corresponding accurate *m*/*z*, a fragmentation pattern was obtained by running the analysis under the targeted MS^2^ mode. The accurate *m*/*z* was assigned to the corresponding Q1 if similar fragmentation patterns were obtained. Eventually, according to MWDB, the metabolites were identified by comparing their mass-to-charge (*m*/*z*) values, retention time, and fragmentation patterns with the standards. The multipeak graph of MRM metabolite detection (figure not shown) exhibited all substances that could be detected in our samples, with each mass spectrum peak of different color representing different metabolites.

The metabolites were quantified by MRM of triple-quadrupole mass spectrometry. In the MRM model, the precursor ions (parent ions) of the target substance were screened by first quadrupole, then made collision by the second quadrupole, which resulted in many fragment ions. Then, through triple-quadrupole, the fragment ions were filtered to choose the characteristic ion, and eliminate nontarget ion interference simultaneously, which made quantitative more accurate. After collecting the mass spectrum data of metabolites from different samples, the mass spectrum peaks of all substances were integrated and corrected [37].

### 3.6. Quality Control

In order to monitor the reproducibility of the analysis process, the mixture of the sample extracts, used as quality control (QC) samples, were injected in every 10 test samples during the measurement. As shown in Figure 1a, the PCA result exhibited that the QC samples (mix1, 2, 3) were distributed in the middle of the 5 group samples, suggesting that the high stability of the instrument provided an important guarantee for data repeatability and reliability.

### 3.7. Data Analysis

Principal component analysis (PCA) was performed using R software (www.r-project.org) with the built-in statistical function prcomp scale = True, meaning unit variance scaling and normalization on raw metabolic data. Then, hierarchical cluster analysis (HCA) of metabolites from different samples was presented through pheatmap within R software. Orthogonal partial least-squares discriminant analysis (OPLS-DA), combined with orthogonal signal correction (OSC) and PLS-DA methods, was performed using the OPLSR.Anal function in MetaboAnalystR package of R software, after the raw data were log-transformed (log2) and mean centering was conducted.

## 4. Conclusions

This study demonstrated the significant effects of storage time on the chemical constituents of oolong teas. A total of 591 metabolites were identified, and of them, 293 metabolites changed significantly during long-term storage. With an increase in storage time, most nongalloylated catechins, tannins including TFs and proanthocyanidins, flavonols and glycosylated flavonols, amino acids and their derivatives, nucleotides and their derivatives, and lots of alkaloids and phospholipids declined, while most fatty acids and organic acids increased, and galloylated catechins, GA, and caffeine were almost stable. Meanwhile, some substances such as chlorogenic acid, theanine, γ-aminobutyric acid, and theophylline were detected at their highest levels in seven-year samples. Both the PCA and chemical changes suggested that approximately seven years (but not an infinite extension) was a special period for oolong tea storage, which would bring about excellent taste. This study also suggests that widely targeted metabolomics combined with multivariate analysis could be applied for quality evaluation and comparison in food science research.

## Supplementary Materials

Table S1: the identification information of total metabolites. Index: ID in MetWare database; Q1 (Da): the molecular weight of parent ions; Q3 (Da): the molecular weight of characteristic fragment ions; Molecular weight: the relative molecular weight of substances; Formula: the molecular formula of substances; Ionization model: lionization model (M+H is the positively charged, M-H is the negatively charged); Compounds: the name of substances; Class I: the first class of substances; Class II: the second class of substances; CAS: the number of substances in CAS; Level: the identification level of substances (A means that the secondary spectral information and the retention time of substances were consistent with the database, while B means that the Q1, Q3, RT, DP and CE of substances were consistent with the database.); 1 Y to 7 Y: the relative contents of substances in corresponding samples; Mix 01 to 03: the relative contents of substances in control samples; cpd-ID: the ID information of substances in KEGG database; kegg-map: the singal path number of substances in KEGG database.

## Sample Availability

The samples of 5 aged Shuixian oolong teas are available from the authors.

## Abbreviations

UPLC-MS/MS, ultra-performance liquid chromatography/tandem mass spectrometry; PCA, principal component analysis; HCA, hierarchical clustering analysis; VIP, variable influence on projection; OPLS-DA, orthogonal partial least-squares discriminant analysis; GA, gallic acid; C, catechin; EC, epicatechin; GC, gallocatechin; EGC, epigallocatechin; EGCG, epigallocatechin-3-gallate; ECG, epicatechin-3-gallate; GCG, gallocatechin-3-gallate; CG, catechin-3-gallate; TFs, theaflavins; TRs, thearubigins; TBs, theabrownines; GABA, γ-aminobutyric acid; 1 y, 1 year; 3 y, 3 years; 7 y, 7 years; 14 y, 14 years; 25 y, 25 years.
